# Overflowing Disparities: Examining the Availability of Litter Bins in New York City

**DOI:** 10.3390/ijerph19095107

**Published:** 2022-04-22

**Authors:** Nadav L. Sprague, Ariana N. Gobaud, Christina A. Mehranbod, Christopher N. Morrison, Charles C. Branas, Ahuva L. Jacobowitz

**Affiliations:** Department of Epidemiology, Mailman School of Public Health, Columbia University, New York, NY 10032, USA; ang2167@cumc.columbia.edu (A.N.G.); cam2376@cumc.columbia.edu (C.A.M.); cm3820@cumc.columbia.edu (C.N.M.); ccb2166@cumc.columbia.edu (C.C.B.); aj2476@columbia.edu (A.L.J.)

**Keywords:** environmental justice, waste management, trash, debris, urban health, environmental health disparities

## Abstract

In the 1980s, activists’ concerns about the disproportionate placements of landfills in low-income communities ignited the environmental justice movement. Today, similar issues of environmental injustice—the limited availability of litter bins across New York City (NYC) neighborhoods—remain unresolved. This study examines the association between NYC neighborhood income and litter bin availability. The NYC Department of Sanitation 2020 Litter Bin Inventory and archival measures of neighborhood composition and socioeconomic status were aggregated within NYC census tract neighborhoods. Multilevel Bayesian conditional autoregressive Poisson models estimated the prevalence rate ratio for counts of litter bins according to median household income in each census tract, accounting for spatial autocorrelation. Bivariate associations identified that census tracts with higher median household income had a greater prevalence of litter bins than census tracts with lower median household income; however, spatial autocorrelation attenuated the relationship between median household income and availability of litter bins. Further research is necessary to identify the spatially structured condition that accounted for the observed effect. The results warrant further investigation of both perceived and actual disparities in litter bin availability.

## 1. Introduction

While many studies have examined the environmental injustice issues surrounding waste management, none have investigated the disparities in litter bin distribution [1]. In the United States, waste management sites are more likely to be placed near Black and low-income communities [1,2]. Environmental health risks are associated with high exposure from working or living near waste management sites, such as landfills, incineration sites, and sites with hazardous waste [1,3,4]. Exposure to waste management sites is associated with adverse birth outcomes [5], respiratory issues [6], all cancers [1], and higher mortality [1]. In the 1980s, civil rights activists’ concerns about the disproportionate placement of landfills in low-income and Black communities sparked the environmental justice movement and related research [7]. 

Today, environmental justice concerns are being voiced about litter bin availability in New York City (NYC) [8]. In August of 2018, the New York City Sanitation Department (DSNY) removed 223 litter bins from Harlem, a neighborhood with a greater proportion of low-income residents [8,9]. Similar to the roots of the environmental justice movement, local residents and leaders raised sanitation and public health concerns associated with the removal of these litter bins [8]. Despite complaints, in July of 2020, DSNY faced budget cuts, resulting in increased concerns by Harlem residents about overflowing litter bins and litter in the streets [10,11].

Some studies have found that increased litter bin availability is associated with decreased litter in the streets [12,13,14]. One study that interviewed residents of Mexico City, Mexico (*n* = 300) found limited litter bin availability to be one of the four primary causes of street litter [13]. Another study conducted a systematic observation of individuals (*n* = 9757) in outdoor public locations across the United States and found that the presence of litter bins reduced individuals’ littering behaviors; in contrast, the presence of street litter increased individuals’ littering behaviors [15,16]. 

Disparities in litter bin availability pose a public health problem, as there are numerous and significant neighborhood health implications to excessive trash and street litter. First, litter attracts rodents, cockroaches, and other pests that may trigger asthmatic or allergic reactions [17,18]. They may also carry vector-borne diseases (such as rat-bite fever, salmonella, and leptospirosis) [17,18,19]. Second, litter is harmful to local flora, fauna, and overall ecological health [20]. Third, some studies suggest that street litter causes injuries such as punctures, tripping, loss of balance or control, and collisions [21]. Fourth, research also suggests that increased neighborhood litter may be associated with increased fear, perceived risk, and actual crime [22,23,24]. Increased street litter has also been linked to a decrease in both neighborhood park use and physical activity for adults [25,26]. Finally, litter in neighborhoods has a negative financial impact on the community, such as reducing home values and detracting potential customers from businesses [27,28]. 

Current concerns about the removal of litter bins from low-income communities in NYC make this research question both relevant and community influenced. This study is exploratory and novel as it is the first to examine environmental injustices associated with the limited availability of street litter bins. We will investigate the prevalence of street litter bins in NYC relative to neighborhood median household income using two models—a Multilevel Bayesian Poisson model and a Multilevel Bayesian model with the addition of a conditional autoregressive random effect. This paper innovatively examines the association between litter bin count and median household income, adjusting for relevant covariates. 

## 2. Theoretical Framework

**Figure 1:**
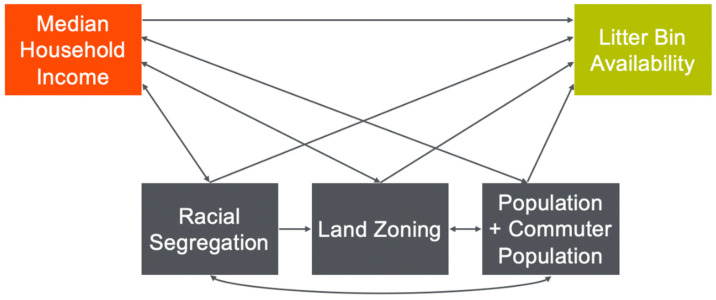
Theoretical framework.

We present our theoretical framework in Figure 1. Low-income communities have historically faced environmental justice issues more than their high-income counterparts, have less social capital, and have less available time to advocate for community-level environmental improvements [29,30,31]. As such, we identify census tract-level median household income as the primary independent variable [1,4,32]. Based on the current literature, we hypothesize that high-income census tracts have greater litter bin availability than low-income census tracts [33,34]. 

We also theorize that median household income may be indirectly associated with litter bin availability through racial segregation, land zoning, population, and commuter population. In the United States, there are well-documented racial disparities in median household income [35]. These racial economic disparities have allowed for the preservation of racial segregation [36]. In turn, the legacy and presence of racial segregation in the US has limited the amount of recourses for certain communities, thus influencing a community’s median household income [36]. As such, we present bidirectional associations (represented by the double-headed arrows) between median household income and racial segregation in Figure 1 [37,38]. Additionally, in the United States, land zoning has either inadvertently or overtly been used to promote economic segregation [39,40]. Therefore, in our theoretical framework, we present bidirectional relationships between median household income and land zoning. Since the 1960s, there have been claims that the misuse of land zoning has contributed to the prevention of equitable access to urban infrastructure and has created clusters of poverty within NYC [41,42]. Thus, it is reasonable to infer that land zoning is associated litter bin availability. There are well documented associations between median household income and population density [39]. We also expect that increases in population density are associated with increases in litter bin availability. 

## 3. Materials and Methods

### 3.1. Study Design

We conducted a cross-sectional analysis to estimate the prevalence of litter bins in NYC, which has a population of approximately 8.4 million people [43]. The unit of analysis was census tract; we included 2101 census tracts in our study. 

### 3.2. Dependent Variable

The main dependent variable was a count of litter bins in NYC census tracts. We obtained litter bin information through NYC Open Data [44]. The DSNY collects data on litter bin location, counts, and type for litter bins owned or tracked by DSNY (i.e., Business Improvement Districts, non-profit organizations, and private companies) via quarterly surveys. The data do not include litter bins owned by other city, state, or federal agencies (including the NYC Department of Parks). For this analysis, we used the 2020 2nd quarter dataset (data collected between April and June 2020). We geocoded litter bins with valid ID numbers in ArcGIS V.10.7.1. All street litter bins with valid ID numbers were successfully geocoded. Litter bin locations were snapped to the nearest roadway section, as denoted in TIGER Line files for roadways in NYC [45]. 

### 3.3. Independent Variable

We considered median household income as the main independent variable and investigated its association with litter bin access. We obtained median household income data on the census tract level from the 2019 American Community Survey (ACS) 5-year estimates [46]. 

Other independent measures included racial segregation, population, commuter-adjusted population, and land use. We measured racial segregation through two different indices: (1) the Index of Concentration at Extremes (ICE) and (2) Massey’s Index of Dissimilarity. The ICE measures racial segregation on a larger scale (i.e., census tracts), whereas the Massey’s Index of Dissimilarity measures racial segregation using a smaller scale (i.e., census block-groups). We calculated the ICE using ACS data by subtracting the total population of non-Hispanic White residents by the population of non-Hispanic Black residents and dividing by the total population. The ICE ranges from −1 to 1, where −1 is a segregated census tract of only non-Hispanic Black residents, 0 is a fully integrated census tract, and 1 is a segregated census tract of only non-Hispanic White residents [47]. Massey’s Index of Dissimilarity measures the percentage of a group’s population that would have to change residence for each neighborhood to have the same percentage of that group as the metropolitan area overall [48]. The index ranges from 0 (complete integration) to 1 (complete segregation). We accessed census tract population data from the 2019 ACS [46]. We measured the number of people living in, working in, or both living and working in each census tract through the commuter-adjusted population. We calculated the commuter-adjusted population by adding the total census tract population with the total number of workers of each census tract and then subtracting the total number of workers that live in that census tract from that value [49]. The total number of workers of each census tract was obtained from the Census Transportation Planning Product’s Census Tract Flows Dataset and the total number of workers that live in that census tract was collected from the 2019 ACS [46,50]. We used county parcel files provided by the US Census Bureau to calculate the percentage of land area that is retail, industrial, and green space within census tracts [45]. 

### 3.4. Statistical Analysis

Multilevel Bayesian Poisson models estimated the prevalence rate ratio for counts of litter bins in each census tract. Model 1 examined the association between litter bin count and median household income, adjusting for relevant covariates. The total length of all roadways (miles) per census tract was included as an offset term to account for the differing sizes of each census tract [45]. Model 2 was specified similarly to Model 1, with the addition of a conditional autoregressive random effect:

$$\log\left(\lambda_{i}\right)=\log\left(e_{i}\right)+{X^{\prime }}_{i}\beta +\theta_{i}$$

$$\exp\left(\theta_{i}\right)\sim \;gamma\left(a,a\right)$$

$$\mathrm{over}\;\mathrm{dispersion}\;\mathrm{parameter}:k=\frac{1}{a}$$

where *λ_i_* is the underlying Poisson mean for the litter bin count at the census tract *i*. *X_i_* represents the covariates and *β* represents the coefficients. *θ_it_* denotes the extra residual term for site *i*.

The conditional autoregressive random effect accounts for spatial dependencies [51,52,53] that arise because nearby census tracts are more likely to have similar values than distal census tracts [54]. As such, Model 2 accounts for the phenomenon that areas close to one another are similar, whereas Model 1 does not take this into account. All statistical analyses were conducted in R and RStudio [55] using the R-INLA package, which provides asymptotic approximations of the Markov Chain Monte Carlo simulation obtained from a full Bayesian procedure. Maps were created using R and RStudio [55] to visually display the test of spatial dependence of litter bin count by census tract in NYC as well as the correlation between count of litter bins and median household income.

## 4. Results

Table 1 presents the distribution of selected characteristics on the census tract level (*n* = 2101). The count of litter bins per census tract ranged from 0 to 117, with a mean of 11.2 litter bins (SD = 13.9). The mean census tract-level population was 4012 (SD = 2181.3) and mean commuter-adjusted population was 3649 (SD = 4455.2). The mean median household income was USD 67,291.90 (SD = USD 33,041.50). The mean ICE was 0.1 (SD = 0.7), suggesting a large variation in racial segregation on the census tract level. The mean dissimilarity index was 0.3 (SD = 0.2), suggesting a smaller variation in racial segregation on the census block groups than the census tracts. 

**Figure 2:**
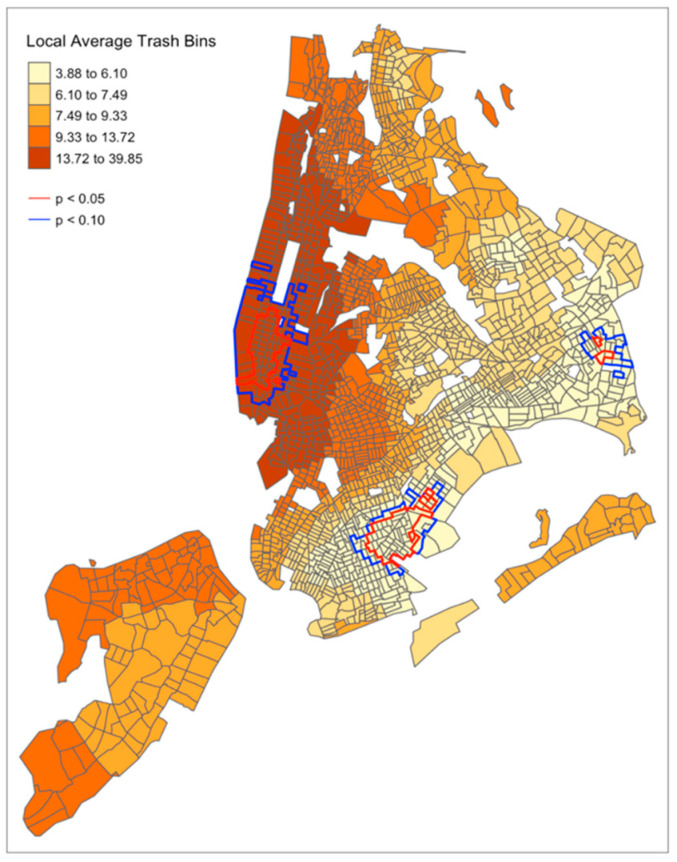
Mapping test of spatial dependence of litter bin count by census tract in New York City.

**Figure 3:**
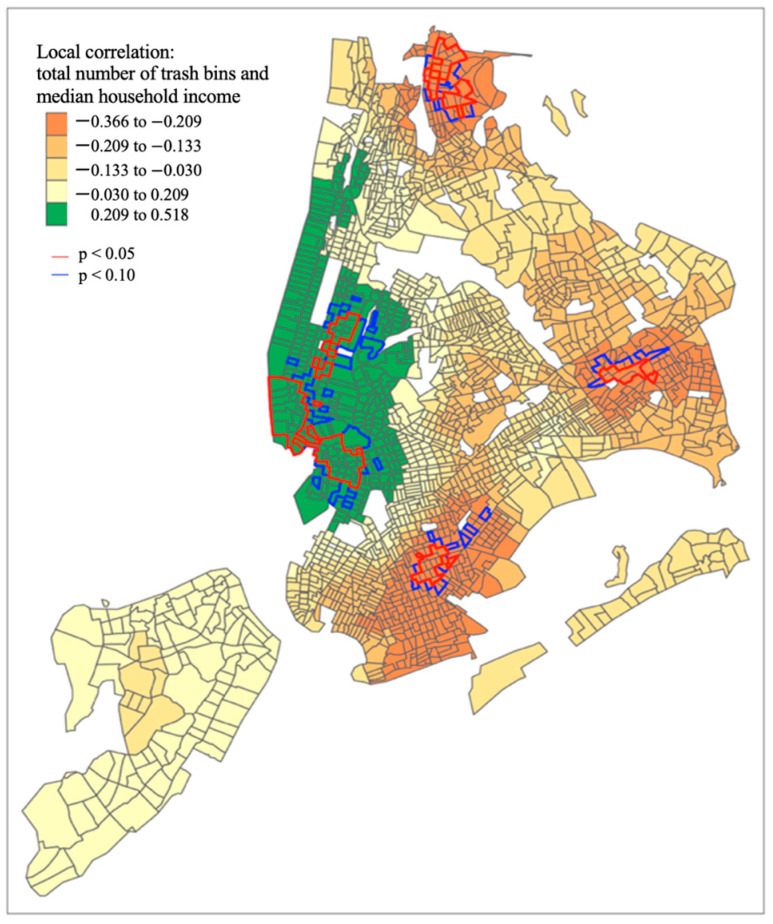
Mapping correlation between count of litter bins and median household income.

Figure 2 depicts the spatial dependence of litter bin count by census tract in NYC. Census tracts with significant spatial autocorrelation at an alpha of 0.05 are outlined in red and census tracts with significant spatial autocorrelation at an alpha of 0.10 are outlined in blue. Figure 3 depicts the correlation between the count of litter bins in a census tract and median household income. There are significant correlations between median household income and litter bin availability in census tracts located in Manhattan, Brooklyn, Queens, and the Bronx, outlined in red at an alpha of 0.05 and outlined in blue at an alpha of 0.10.

The model results can be found in Table 2. In Model 1, we observed a positive association between median household income and litter bin availability (prevalence rate ratio (PRR) = 1.13; 95% CI: 1.11–1.15). A USD 10,000 increase in census tract median household income was associated with a 13% increase in the count of litter bins per census tract when adjusting for other covariates. In Model 2, median household income was no longer significantly associated with litter bin count once spatial dependencies were accounted for (PRR = 0.95; 95% CI = 0.88–1.03).

## 5. Discussion

This exploratory analysis examined the potential inequities in street litter bin availability in NYC census tracts. The bivariate associations identified that census tracts with higher median household income had a greater prevalence of litter bins than census tracts with lower median household income. However, in fully adjusted models that control for theoretically relevant covariates and spatial dependencies, median household income was not associated with litter bin availability.

Our findings show that there are more litter bins in high-income areas than low-income ones. However, this relationship was attenuated when controlling for relevant covariates and the spillover influence of neighborhoods that were close to one another: spatial autocorrelation. As such, this analysis may suggest that NYC equitably distributes litter bins throughout the city. However, the explanation of spatial autocorrelation attenuating the relationship between median household income and litter bin availability may be more nuanced. The disparities of litter bin availability found in our initial models may be an effect of larger, more structural income-related disparities in New York City. In the United States, both median household income and litter bin availability are spatially dependent [56]. In our study, we found that a spatially structured condition accounts for the observed effect of disparities in access to litter bins. Similarly, other spatial analyses have documented the clustering of both high-poverty and low-poverty counties throughout the United States [57], meaning that spatially structured conditions also partially account for disparities in median household income. Research suggests that governmental policies (e.g., taxation, zoning, and minimum floor space requirements) induce and sustain the spatial sorting and clustering by income [56]. 

Additionally, commuter population appeared to have the strongest relationship with litter bin availability in both models. For large metropolitan cities in the United States, including New York City, low-income residents tend to have longer commute times than higher-income residents [58,59]. In these large cities, the higher-income areas have higher commuter populations than the lower-income areas [59]. As such, disparities in litter bin availability may be an effect of the relationship between neighborhood median household income and commuter population. More research is warranted to understand the relationship between neighborhood income, commuter population, and litter bin availability. 

While this analysis suggests that New York City may equitably distribute litter bins, it is also important to note the importance of residents’ environmental health perceptions. Harlem residents clearly voiced concerns about overflowing trash in their neighborhoods [8,10]. A large body of evidence suggests that perceptions of environmental exposures may have equal or greater effects on the outcome than the actual exposure [60,61,62,63,64,65]. For example, one study that investigated the perceived and actual weather exposures on adults’ walking behavior found that the effect of the actual exposure (extreme temperatures) was dependent on the perceived exposure (individual’s perceptions on extreme weather being a barrier to walking) [64]. Another study that examined the effects of perceived and actual indoor and outdoor air quality found that environmental exposure perceptions had a significant impact on health outcomes, even in areas with the same levels of air pollution [65]. While it quantitatively appears that New York City is equitably distributing litter bins, the perceptions of litter bin availability at the local level are equally important. Additional qualitative and quantitative research is warranted to understand community members’ perceptions on litter bin availability disparities, as the perceptions may have an equal or greater effect than what can be measured in terms of exposure. Local authorities should also hold community forums to understand community members’ concerns and collaboratively identify appropriate solutions.

This study should be interpreted with its limitations in mind. First, the DSNY dataset did not include litter bins that were owned by other city, state, or federal agencies. These additional litter bins that are not included may or may not be equally distributed across NYC. Future studies should include litter bins tracked by the DSNY dataset, as well as other city, state, and federal agencies. Second, the data used in this analysis were cross-sectional as the only publicly available data were from the second quarter of 2020. Every year the DSNY’s budget fluctuates and over time the distribution of litter bins may also fluctuate. DSNY only publicly provides the most up to date data; however, future investigations should be longitudinal in nature. Next, we were unable to distinguish litter bin type due to limited data publicly provided by DSNY. Having this information is important as there is evidence that litter bins with lids or a covering are more effective in reducing street litter than litter bins without [66]. Finally, this study only examined litter bin availability in NYC and cannot be generalized beyond that region. Future studies should be conducted in other metropolitan areas, both nationally and globally, to understand how they compare to NYC. 

The analyses in this study were preliminary, and therefore future studies are required to further understand the multidimensional and complex dimensions of litter bin access disparities. For example, future studies should explore the health outcomes and consequences of the distribution of litter bins in NYC statistically. Additional research directions include investigating environmental health perceptions of litter bin availability through conducting community focus groups and then developing a larger-scale questionnaire based on the focus groups’ results. However, future research is required before we would be able to draw conclusions on what efforts can be undertaken by multiple actors in municipal solid waste management to achieve sustainable and equitable development in NYC.

## 6. Conclusions

Residents and community leaders have voiced concern about the removal of litter bins in low-income NYC neighborhoods. We observed that median household income was significantly associated with the availability of litter bins in our initial models that did not account for spatial autocorrelation; however, this relationship was no longer significant after accounting for spatial autocorrelation. As such, disparities in litter bin availability may be an effect of structural income-related disparities in New York City, such as inequitable governmental housing policies and economic disparities in commuter time. Further, while New York City may equitably distribute litter bins, perceptions on environmental exposures may have equal or greater health effects than the actual environmental exposure. As such, future research should investigate environmental perceptions of litter bin availability on health. 

## Figures and Tables

**Table 1:** Descriptive statistics for 2101 census tracts in New York City *.

Variable	Mean	Standard Deviation	Minimum	Maximum
Litter Baskets	11.2	13.9	0.0	117.0
Median Household Income	67,291.9	33,041.5	9939.0	250,001.0
Population	4012.0	2181.3	36.0	28,272.0
Commuter Population	3649.0	4455.2	78.0	52,767.0
ICE **	0.1	0.7	−1.0	1.0
Dissimilarity Index	0.3	0.2	0.0	1.0
% Retail	0.4	3.7	0.0	96.8
% Industrial	0.3	3.4	0.0	69.4
% Green space	4.1	10.5	0.0	100.0

* Data are from 2019 ACS, ** ICE = Index of Concentration at Extremes.

**Table 2:** Multilevel Bayesian conditional autoregressive Poisson models estimating the prevalence rate ratio for counts of litter bins.

Variable	Model 1 ^1^			Model 2 ^2^		
	PRR	95% CI		PRR	95% CI	
Median Household Income	**1.13**	**1.11**	**1.15**	0.95	0.88	1.03
ICE *	**1.08**	**1.06**	**1.09**	0.92	0.82	1.03
Dissimilarity Index	**1.08**	**1.06**	**1.09**	1.04	0.99	1.09
Population	**1.08**	**1.07**	**1.09**	1.04	0.98	1.10
Commuter Population	**1.26**	**1.25**	**1.27**	**1.14**	**1.06**	**1.22**
% Retail	0.99	0.98	1.00	1.01	0.96	1.06
% Industrial	**0.80**	**0.78**	**0.82**	**0.88**	**0.84**	**0.93**
% Green space	**0.76**	**0.75**	**0.77**	**0.79**	**0.75**	**0.83**

Bold numeric values indicate statistical significance (*p* < 0.05). * ICE = Index of Concentration at Extremes. ^1^ Model 1 estimated the prevalence rate ratio for counts of litter bins by median household income in each census tract, adjusting for confounders. ^2^ Model 2 estimated the prevalence rate ratio for counts of litter bins by median household income in each census tract, adjusting for covariates and accounting for spatial dependencies.

## Data Availability

Publicly available datasets were analyzed in this study. These data can be found here: https://opendata.cityofnewyork.us/ (accessed on 31 December 2020).
